# The central role of DNA damage in immunosenescence

**DOI:** 10.3389/fragi.2023.1202152

**Published:** 2023-07-03

**Authors:** Loren Kell, Anna Katharina Simon, Ghada Alsaleh, Lynne S. Cox

**Affiliations:** ^1^ Department of Biochemistry, University of Oxford, Oxford, United Kingdom; ^2^ Botnar Institute for Musculoskeletal Sciences, Nuffield Department of Orthopaedics, Rheumatology and Musculoskeletal Sciences (NDORMS), University of Oxford, Oxford, United Kingdom; ^3^ The Kennedy Institute of Rheumatology, Nuffield Department of Orthopaedics, Rheumatology and Musculoskeletal Sciences (NDORMS), University of Oxford, Oxford, United Kingdom; ^4^ Max Delbrück Center for Molecular Medicine, Berlin, Germany

**Keywords:** ageing, immunosenescence, senescence, immunology, DNA repair, DNA damage

## Abstract

Ageing is the biggest risk factor for the development of multiple chronic diseases as well as increased infection susceptibility and severity of diseases such as influenza and COVID-19. This increased disease risk is linked to changes in immune function during ageing termed immunosenescence. Age-related loss of immune function, particularly in adaptive responses against pathogens and immunosurveillance against cancer, is accompanied by a paradoxical gain of function of some aspects of immunity such as elevated inflammation and increased incidence of autoimmunity. Of the many factors that contribute to immunosenescence, DNA damage is emerging as a key candidate. In this review, we discuss the evidence supporting the hypothesis that DNA damage may be a central driver of immunosenescence through senescence of both immune cells and cells of non-haematopoietic lineages. We explore why DNA damage accumulates during ageing in a major cell type, T cells, and how this may drive age-related immune dysfunction. We further propose that existing immunosenescence interventions may act, at least in part, by mitigating DNA damage and restoring DNA repair processes (which we term “genoprotection”). As such, we propose additional treatments on the basis of their evidence for genoprotection, and further suggest that this approach may provide a viable therapeutic strategy for improving immunity in older people.

## 1 Introduction

The past century has seen a marked increase in overall human lifespan (Oeppen and Vaupel, 2002; Dong et al., 2016), largely arising from improvements in diet, lifestyle, sanitation, and healthcare. However, this increase in life expectancy has not been met by an increase in healthy life expectancy, with ageing being the biggest risk factor for developing chronic diseases and multimorbidity (Barnett et al., 2012). Older people experience an overall decline in their responses to immune challenges such as vaccination (Lord, 2013; Wagner et al., 2018), resilience against novel pathogens [e.g., SARS-CoV2 (O'Driscoll et al., 2021; Hou et al., 2022)], and diminished antigen-specific memory responses (Vukmanovic-Stejic et al., 2018) together with reduced immunosurveillance for cancer neoantigens (Ovadya et al., 2018). Age-related immune dysfunction is also associated with an increase in low-grade inflammation, termed inflammageing (Franceschi et al., 2000). These maladaptive changes in immunity are broadly termed immunosenescence [reviewed in (Teissier et al., 2022)]. Despite major advances in the field, there are still significant gaps in our understanding of the causes of immunosenescence, and of how immunosenescence underpins the age-related increase in susceptibility to and severity of infections. As the recent COVID-19 pandemic highlighted, new therapies to alleviate immunosenescence are urgently needed, and our increasing understanding of ageing biology may provide such therapies (Cox et al., 2020).

Biological underpinnings of the ageing process have been encapsulated in the hallmarks of ageing (Lopez-Otin et al., 2013; Schmauck-Medina et al., 2022; Lopez-Otin et al., 2023). Immunosenescence is associated with many of these ageing hallmarks, including genome instability, while stromal cellular senescence and altered intercellular communication contribute to inflammageing (Aiello et al., 2019). However, though many ageing hallmarks overlap with immunosenescence, it is still unclear whether the hallmarks causally drive age-related immune decline and whether they act in a cell autonomous manner. For example, the proportion of cells which have undergone proliferative arrest and become senescent increases in both haematopoietic (immune) and non-haematopoietic compartments during ageing (Lopez-Otin et al., 2013; Tuttle et al., 2020; Mittelbrunn and Kroemer, 2021). Although many triggers for cellular senescence have been identified (van Deursen, 2014), there is accumulating evidence that these converge upon, and are further driven by, the ageing hallmark of genome instability and persistent DNA damage (Yousefzadeh et al., 2021a; Schumacher et al., 2021). Indeed, markers of DNA damage are observed to increase in the immune system during ageing, but until recently it was unknown whether this was either causal, or secondary to other age-related organismal changes. In support of a causative role, recent studies have demonstrated that inducing persistent DNA damage in haematopoietic cells alone is sufficient to recapitulate ageing-induced immunosenescence and organismal ageing (Desdin-Mico et al., 2020; Yousefzadeh et al., 2021b). While we do not know whether senescence of other cell types would have had this effect, these recent findings suggest that DNA damage in the immune system may be causal in precipitating immunosenescence and accelerating whole body ageing.

In this review, we explore the role of DNA damage in relation to age-related dysfunction of the immune system. We consider how DNA damage contributes to cellular senescence and accelerated ageing in haematopoietic cells and non-haematopoietic cells, and examine the causes of increased DNA damage in immune cells during ageing, focusing on T cells. We further discuss how senescence in non-blood-forming cells impairs immunity in older individuals. Finally, we suggest that therapies targeting immunosenescence, particularly through restoring genome stability (which we call “genoprotective” therapies), may be a promising approach for improving immunity in older people and reducing age-related risk of both chronic non-communicable and infectious diseases.

## 2 DNA damage and cellular senescence

### 2.1 DNA lesions and repair pathways

Endogenous and exogenous factors can lead to a variety of different types of genome lesions, on one or both strands of the DNA, and that occur at scales ranging from individual bases to much larger tracts of the genome (Huang and Zhou, 2021). Endogenous sources of DNA damage include reactive oxygen species (ROS), normal by-products of oxidative metabolism which are generated at abnormally high levels by dysfunctional mitochondria [a hallmark of ageing (Lopez-Otin et al., 2013)]; ROS can induce oxidative DNA damage and lead to DNA replication errors that require DNA excision repair pathways to correct. Exogenous UV light can induce lesions including (6–4) photoproducts and cyclobutane pyrimidine dimers (i.e., covalently linked adjacent cytosine or thymidine bases on one DNA strand), while interstrand crosslinks can be formed by crosslinking agents such as mitomycin C. Other exogenous sources of DNA damage, such as ionising radiation, or radiomimetic genotoxins such as etoposide or bleomycin, can induce single- or double-strand breaks (SSBs or DSBs) i.e., breaks in one or both of the DNA strands of a duplex (Mason and Cox, 2012).

**FIGURE 1:**
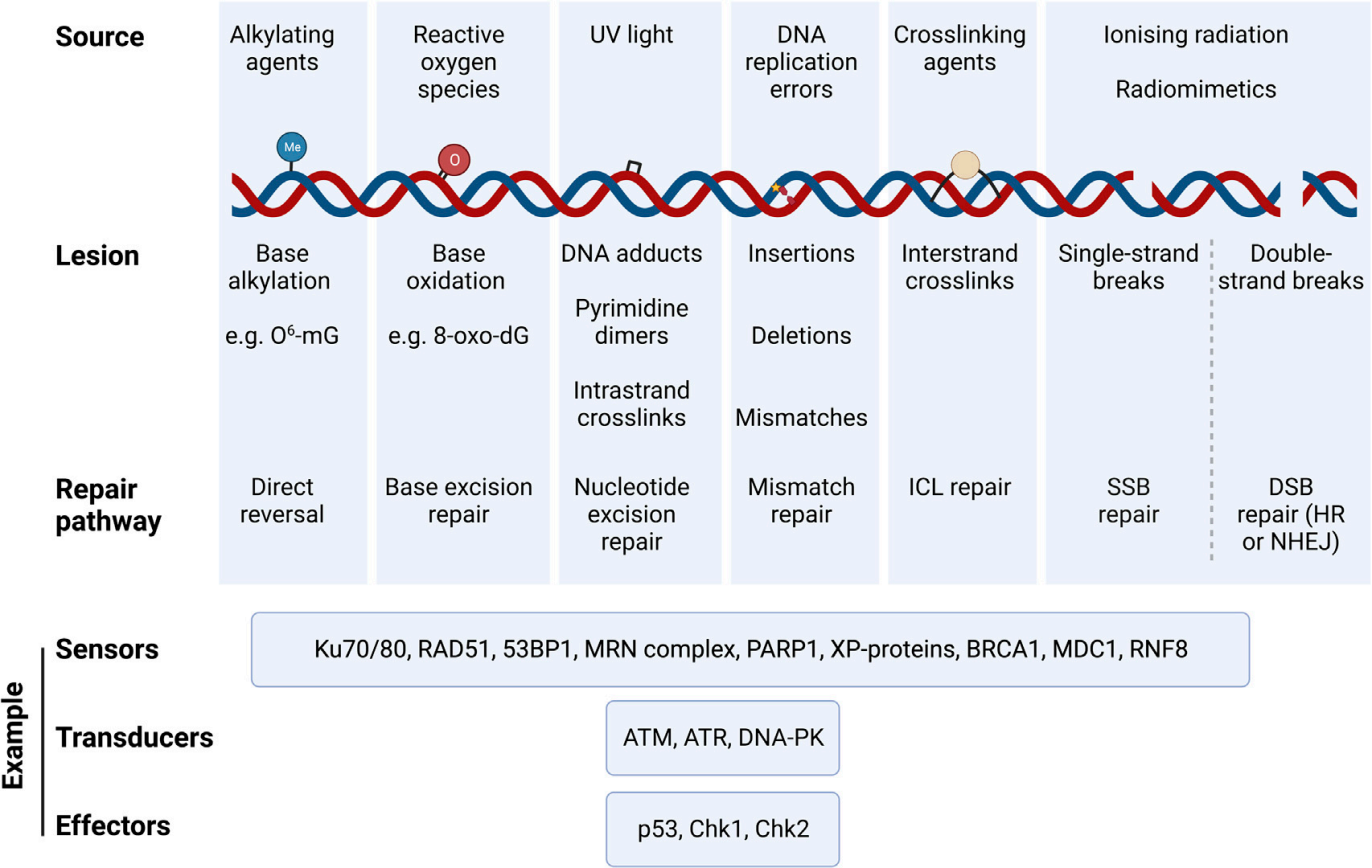
**Types of DNA damaging agents, resulting lesions and canonical repair pathways that correct them.** DNA experiences a variety of genotoxic insults from both endogenous and exogenous sources. These result in specific lesions that can be repaired via specific DNA repair pathways that are triggered by the DNA damage response (DDR). Damaged DNA is sensed, signalled, and repaired through a cascade of signalling mechanisms elicited by DNA sensors, tranducers, and effectors, which are specific to the DNA lesion type and repair pathway. Examples of such DDR proteins are illustrated. Abbreviations: O6-mG, O-6-methylguanine; 8-oxo-dG, 8-oxo-2′-deoxyguanosine; ICL, interstrand crosslink; HR, homologous recombination; NHEJ, non-homologous end joining; 53BP1, p53·-binding protein 1; MRN complex, Mre11/Rad50/Nbsl; PARP1, Poly [ADP-ribose] Polymerase 1; XP-proteins, Xeroderma Pigmentosum proteins; BRCA1, breast cancer gene 1; MDC1, Mediator of DNA Damage Checkpoint 1; RNF8, Ring Finger Protein 8; ATM, Ataxia-Telangiectasia Mutated; ATR, Ataxia Telangiectasia and Rad3-related; DNA-PK, DNA Protein Kinase complex; Chk1, Checkpoint Kinase 1; Chk2, Checkpoint Kinase 2.

Distinct pathways have evolved to repair the different types of DNA lesions [reviewed recently in (Huang and Zhou, 2021)]. These include i) direct reversal of the damage, ii) base excision repair (BER), iii) nucleotide excision repair (NER) for bulkier adducts and complicated lesions, iv) mismatch repair (MMR), v) interstrand crosslink (ICL) repair, vi) single-strand break (SSB) repair and vii) double-strand break (DSB) repair, chiefly by either homologous recombination (HR) or non-homologous end joining (NHEJ). Damage to the DNA triggers the DNA damage response (DDR), which involves a tightly coordinated recruitment of damage sensors, transducers and effectors that transiently arrest the cell cycle, and drive cell fate decisions to either DNA repair, apoptosis, or senescence. Figure 1 provides a summary of DNA lesion types and their canonical repair mechanisms. Indeed, the presence of DNA damage can be probed by measuring the activation and/or localisation of proteins involved in the DDR pathways, such as γH2AX (histone variant H2AX phosphorylated at Ser^139^), p53-binding protein 1 (53BP1) (Huang and Zhou, 2021), and the phosphorylation of key DDR master regulators, ATM (at Ser^1981^) and ATR (at Thr^1989^). It is possible to probe for specific DNA lesion epitopes [e.g., 8-oxo-dG – e.g., (Sanderson and Simon, 2017)], while DNA fragmentation due to SSBs and DSBs can be directly measured using single cell gel electrophoresis (the comet assay); inverse comet assays can instead provide a readout of extent of interstrand crosslinking (Swift et al., 2020). Overall, cells have evolved a plethora of mechanisms to repair DNA damage; whether these are implemented successfully plays a crucial role in determining cell fate.

### 2.2 Cellular senescence

Cellular senescence, a state of permanent proliferative arrest accompanied by a host of morphological, metabolic and functional changes, was originally discovered during long-term *in vitro* culture of human lung fibroblasts (Hayflick and Moorhead, 1961). Senescent cells accumulate in tissues during normal ageing (van Deursen, 2014; Tuttle et al., 2020) and increased senescent cell burden is a hallmark of ageing (Lopez-Otin et al., 2023). Senescence has been shown to be a causative factor in ageing, contributing to age-related morbidity and mortality – for instance, transplanting senescent cells into mice accelerates physical frailty and shortens lifespan (Xu et al., 2018). Moreover, deletion of senescent cells via genetic (Baker et al., 2016) or pharmacological means [e.g., fisetin (Yousefzadeh et al., 2018), or dasatinib + quercetin, “D + Q” (Xu et al., 2018)] increases median lifespan and ameliorates a range of age-related conditions in mice, thus extending healthspan. Importantly, such senolytic treatment also improved survival following coronavirus infection (Camell et al., 2021; Cox and Lord, 2021), potentially through reducing inflammatory signalling; whether such treatment also improves adaptive immunity is not yet clear. Cellular senescence therefore drives ageing and inflammation.

Although no unique biomarkers for senescence have yet been described, it is associated with high expression of the tumour suppressor p53 (and also at times p-Rb), and the cyclin kinase inhibitors *CDKN1A* (p21^Cip1^) and *CDKN2A* (p16^INK4a^) which establish and maintain proliferative arrest (van Deursen, 2014). Features of senescent cells include stabilised actin stress fibres (Walters et al., 2016), increased activity of lysosomal senescence-associated β-galactosidase (SAβG) at suboptimal pH 6.0 (Dimri et al., 1995), enlarged nucleoli (Buchwalter and Hetzer, 2017), chromatin rearrangements [e.g., SAHFs (Narita et al., 2003)], nuclear lamina breakdown leading to cytoplasmic chromatin fragments [CCFs (Ivanov et al., 2013)] and persistent expression of DNA damage markers such as γH2AX and 53BP1 (Rodier et al., 2011). Senescent cells possess a unique secretome consisting of inflammatory cytokines, matrix-remodelling proteins, and extracellular vesicles, collectively termed the senescence-associated secretory phenotype (SASP) (Coppe et al., 2008; Rodier et al., 2009). Senescence markers in the immune system are similar to those in other cell types, and a variety of tools, including flow cytometry, has proven useful in identifying senescent immune cells (Table 1) (Zhou et al., 2021).

**TABLE 1:** Characteristics of senescent T cells.

Phenotype	Description	Evidence for phenotype in senescent T cells during human ageing	
		CD4^+^ T cells	CD8^+^ T cells
CD27^−^CD28^−^	Stimulatory co-receptors CD27 and CD28 are progressively lost during differentiation from naïve to memory T cell subsets	↑ %CD27^−^CD28 Moro-García et al. (2013)	↑ %CD27^−^CD28^−^ Plunkett et al. (2007)
			↑ %CD27^−^CD28 Henson et al. (2009)
CD27^−^CD45RA^+^ (T_EMRA_)	CD27 and CD45RA define naïve, T_N_ (27^+^RA^+^), central memory, T_CM_ (27^+^RA^−^), effector memory, T_EM_ (27^−^RA-), and senescent, T_EMRA_ (27^−^RA^+^)	↑ %T_EMRA_ Libri et al. (2011)	↑ %T_EMRA_ Callender et al. (2020)
		↑ %T_EMRA_ Callender et al. (2020)	↑ %T_EMRA_ Riddell et al. (2015)
SAβG^high^	Senescence-associated β-galactosidase	↑ % SAβG^high^ Martinez-Zamudio et al. (2021)	↑ %SAβG^high^ Martinez-Zamudio et al. (2021)
KLRG1^+^	Killer Cell Lectin-like Receptor G1, co-inhibitory receptor	T_EMRA_ Di Mitri et al. (2011)	T_EMRA_ Henson et al. (2014)
			T_EMRA_ Henson et al. (2015)
			T_EMRA_ Pereira et al. (2020)
			CD27^−^CD28 Henson et al. (2009)
CD57^+^	Terminal differentiation marker	T_EMRA_ Libri et al. (2011)	T_EMRA_ Henson et al. (2014)
			T_EMRA_ Henson et al. (2015)
			T_EMRA_ Pereira et al. (2020)
			Old donor pan-CD8^+^ Phadwal et al. (2012)
NKR^+^	Natural Killer Receptors	Old donor pan-CD4^+^ Alonso-Arias et al. (2011)	T_EMRA_ Pereira et al. (2020)
↑ Cytokine and cytotoxic granule production	Contributor to cytotoxic and inflammatory nature of senescent T cells, e.g., IFNγ, TNFα, perforin, granzyme B	T_EMRA_ Libri et al. (2011)	T_EMRA_ Henson et al. (2014)
			T_EMRA_ Henson et al. (2015)
			T_EMRA_ Callender et al. (2018)
↑ Sestrins	Sestrin-MAPK activation complex (sMAC) drives maladaptive T cell functions and NKR-mediated cytotoxicity	CD27^−^CD28^−^ Lanna et al. (2017)	CD27^−^CD28^−^ Pereira et al. (2020)
↓ autophagy	Decreased autophagic flux	Old donor pan-CD4^+^ Bharath et al. (2020)	CD57^+^ Phadwal et al. (2012)
			T_EMRA_ Henson et al. (2014)
			Old donor vaccine-specific cells Alsaleh et al. (2020)
↓ proliferative ability		T_EMRA_ Libri et al. (2011)	T_EMRA_ Henson et al. (2014)
			T_EMRA_ Henson et al. (2015)
			CD27^−^CD28^−^ Plunkett et al. (2007)
↓ telomerase activity		T_EMRA_ Di Mitri et al. (2011)	T_EMRA_ Henson et al. (2014)
			T_EMRA_ Henson et al. (2015)
			CD27^−^CD28^−^ Plunkett et al. (2007)
↓ telomere length		T_EMRA_ Di Mitri et al. (2011) (T_EMRA_ have shorter telomeres than T_N_)	CD27^−^CD28^−^ Plunkett et al. (2007)
			Old donor pan-CD8^+^ and T_EMRA_ Sanderson and Simon (2017)
			T_EMRA_ shorter than T_N_ Riddell et al. (2015)
↑ γH2AX		T_EMRA_ Di Mitri et al. (2011)	T_EMRA_ Henson et al. (2014)
		CD27^−^CD28^−^ Lanna et al. (2014)	T_EMRA_ Henson et al. (2015)
		T_EMRA_ Callender et al. (2020)	T_EMRA_ Callender et al. (2020)
↑ p-ATM (Ser^1981^)	Phosphorylation/activation of DDR	CD27^−^CD28^−^ Lanna et al. (2014)	
↑ 53BP1^+^	p53-binding protein 1, DNA damage sensor		SAβG^high^ Martinez-Zamudio et al. (2021)
↑ p-p53 (Ser^15^)	Phosphorylation/activation during DDR		T_EMRA_ Callender et al. (2020)
↑ ROS	Reactive Oxygen Species	CD27^−^CD28^−^ Lanna et al. (2014)	T_EMRA_ Henson et al. (2014)
			Old donor pan-CD8^+^ Sanderson and Simon (2017)
↑ Dysfunctional mitochondria		Old donor pan-CD4^+^ Bharath et al. (2020)	T_EMRA_ Henson et al. (2014)
			T_EMRA_ Callender et al. (2020)

### 2.3 DNA damage drives cellular senescence and accelerated ageing

A variety of senescence inducers have been characterised, including oncogene activation leading to oncogene-induced senescence (OIS) and some viruses, such as SARS-CoV2, leading to virus-induced senescence (VIS) (Lin et al., 1998; Lee et al., 2021); major DNA damage is also a trigger leading to senescence, and persistent DNA damage is often a feature of other senescence types (Fumagalli et al., 2014). Indeed, an imbalance between DNA damage and repair is a primary driver of cell cycle proliferative arrest resulting in one of three key cell fates: persistent arrest, cell death, or cell senescence. How this imbalance arises depends on the type of damage signal involved.

In replicative senescence (RS), the type of cell senescence thought to arise most commonly during normal ageing, damage persistence is a consequence of telomere attrition (Harley et al., 1990). Loss of telomeric DNA at every round of DNA replication gradually erodes telomeres until they become critically short, which impairs the stabilisation of the protective shelterin complex (Rossiello et al., 2014). Uncapping of eroded telomeres is sensed as a DSB, triggering a persistent DDR and permanent exit from the cell cycle. The shortening of telomeres towards this critical length after each mitotic cell cycle therefore fundamentally limits the replicative lifespan of cells, i.e., drives replicative senescence. Maintaining telomere length and capping is therefore important for chromosomal stability and to prevent early onset of senescence. Indeed, ectopic expression of telomerase, which extends telomeres, overcomes premature senescence phenotypes in progeroid Werner syndrome cells *in vitro* (Wyllie et al., 2000), while experimental telomerase reactivation promotes tissue rejuvenation in mice (Jaskelioff et al., 2011). Unlike most somatic cells where telomerase is repressed, adaptive immune T and B cells can upregulate telomerase expression, which prolongs their replicative potential; this is likely to be an evolved response to permit multiple rounds of clonal proliferation upon antigenic stimulation without inducing replicative exhaustion (Akbar et al., 2016). Remarkably, T cells from an immunised mouse could be transferred and expanded in 51 successive recipient mice, over 10 years, without showing any proliferative dysfunction or telomere shortening (Soerens et al., 2023). However, even where telomere length has not reached the point of shelterin destabilisation, DNA damage – particularly 8-oxo-dG – within the telomeric regions is effective at signalling for cell cycle arrest and senescence (von Zglinicki, 2000). Stress-induced premature senescence (SIPS) can occur, e.g., in response to oxidising environments and can result in telomere oxidation. Notably up to half of all DNA damage foci (as measured by a combination of immunofluorescence and ChIP) have been reported to localise at telomeres (Hewitt et al., 2012) suggesting either that telomeres are particularly prone to damage, or that their heterochromatic nature precludes effective repair (Fumagalli et al., 2012; von Zglinicki, 2002). Telomeric vulnerability due to their high guanine content remains the most likely explanation.

As well as oxidative damage, exposure to genotoxic agents can drive DNA damage-induced cellular senescence (DIS). Following transient cell cycle arrest immediately after DNA damage, in cases where DNA lesions are successfully repaired (Figure 1), the cell may re-enter the proliferative cell cycle. However, in situations where the damaging agent persists, where the lesion burden is high, or where the cell is unable to repair the DNA lesions (e.g., through loss of function of critical DNA repair factors), persistent DDR signalling can result in irreversible cell cycle arrest and DIS. DIS is considered to have evolved as a tumour-suppressive strategy to limit cancerous transformation in the presence of a high lesion burden (van Deursen, 2014).

DNA damage is therefore well established as a driver of senescence. It is also strongly associated with pathology during ageing (Schumacher et al., 2021). Indeed, the majority of human premature ageing (progeroid) syndromes (e.g., Werner syndrome, Rothmund-Thomson syndrome, Bloom syndrome, Cockayne’s syndrome, Fanconi’s anaemia, and Ataxia-Telangiectasia) result from mutations in DNA repair factors (WRN, RECQL4, BLM, CSB, FANC proteins and ATM respectively); patients show premature ageing across multiple organs often accompanied by greatly elevated cancer incidence, and patient-derived cells senesce prematurely in culture (Cox and Faragher, 2007). In Hutchinson-Gilford progeria, where the driver mutation occurs in the lamin A/C gene, i.e., not a DNA repair factor, the resulting instability of the nuclear lamina leads to significant genome instability and DNA breakage (Liu et al., 2005). Hence progeroid syndromes provide strong evidence that DNA damage is a major driver of senescence and ageing (Yousefzadeh et al., 2021a).

Further to DNA damage being an inducer of senescence, unrepaired DNA lesions propagate further cellular dysfunction and hallmarks of ageing, thereby positioning DNA damage as a major node in senescence and ageing (Schumacher et al., 2021). For example, free DNA ends at sites of damage are recognised by poly [ADP-ribose] polymerase 1 (PARP1) which uses NAD^+^ as a substrate for sensing the damaged DNA (Yoshino et al., 2018). Continuous and persistent DNA damage therefore leads to increased PARP1 activity and depletion of NAD^+^ stores, resulting in reduced function of other NAD^+^-requiring enzymes such as the sirtuins, a family of NAD-dependent deacetylases, some of which are important regulators of chromatin organisation and mitochondrial health, associated with longevity (Fang et al., 2014). Maintaining sirtuin activity limits senescence: indeed, the age-related loss of SIRT6 activity results in heterochromatin decompaction and expression of retrovirus cDNA in the cytosol, triggering innate cGAS-STING signalling and an interferon-induced SASP response (De Cecco et al., 2019; Simon et al., 2019). Similarly, loss of sirtuin activity in DNA repair-deficient models [e.g., XPA (Fang et al., 2014)] demonstrate that DNA damage can induce mitochondrial dysfunction and additional ROS production, and that ROS is requisite for further deepening senescence (Passos et al., 2010). DNA damage can thus drive a positive feedback mechanism to propagate further dysfunction and senescence.

Since DNA damage is an important trigger and propagator of senescence and ageing pathology, supporting and enhancing cells’ ability to repair DNA lesions and limit senescence may be a valuable strategy for promoting healthy ageing.

## 3 Immunosenescence

Immunosenescence describes organism-level age-related dysfunction of both innate and adaptive immunity, which leads to increased susceptibility to pathogens [most recently exemplified by the COVID-19 pandemic (Cox et al., 2020)], poor vaccine responses (Lord, 2013), elevated cancer risk, and increased autoimmunity (Goronzy and Weyand, 2012). Additionally, immunosenescence is associated with a low-grade inflammatory environment that is generated, at least in part, by the SASP of senescent cells of both immune and non-immune origin (Teissier et al., 2022). Indeed, immunosenescence is partially underpinned by the age-related changes in immune cells, leading to a variety of dysfunctional phenotypes, several of which are characteristic of senescence (Zhou et al., 2021). However, not all age-related changes to immune cells are indicative of cellular senescence - for example, T cells with an exhausted phenotype accumulate with age and/or chronic antigenic stimulation, such as CMV exposure (Fletcher et al., 2005). Furthermore, the age-related shifts in other immune cell subsets, particularly with a bias from anti-inflammatory to pro-inflammatory cells, may contribute to immune dysfunction consistent with immunosenescence (Marquez et al., 2020).

Here we discuss the unique function of physiological DNA damage in lymphocytes. We then discuss how persistent immune DNA damage is associated with ageing and immunosenescence, with a particular focus on T cell senescence. We further describe the known sources of T cell DNA damage, and how genotoxic stress may cause the maladaptive phenotypes of senescent T cells that promote immunosenescence. Finally, we discuss how the senescence of non-haematopoietic cells can suppress adequate antigen-specific responses and thus reduce older people’s ability to respond effectively to neoantigens, leading to risk of severe illness or death from infectious disease.

### 3.1 DNA damage in the immune system

#### 3.1.1 ‘Intentional’ DNA lesions generate receptor diversity

DNA damage within the immune system is not always deleterious. In fact, the introduction of DNA lesions plays an evolved role in generating receptor diversity in the B and T cells of the adaptive immune system; such physiological genome alterations do not trigger the irreversible cell cycle arrest characteristic of cell senescence (Gullickson et al., 2022). V(D)J recombination, employed by both cell types, relies on the induction of DSBs that allow the rearrangement of individual Variable, Diversity, and Joining segments to produce a unique repertoire of immune receptors. B cell receptor genes undergo the critical processes of somatic hypermutation and class-switch recombination, both of which require the introduction of DNA lesions by the enzyme activation-induced cytidine deaminase, and modification by canonical DNA repair pathways and factors - including DNA-PK, Ku, Artemis, DNA ligase IV, and ERCC4 as well as recombination-inducing RAG proteins (Gellert, 2002). Crucially, loss of key DNA repair proteins, particularly those involved in DSB repair, leads to severe combined immunodeficiency characterised by a reduction of T and B cell populations and poor responses to immune challenge (Gullickson et al., 2022). Maintaining proper DNA damage and repair processes is therefore central for normal immune function.

#### 3.1.2 Immune DNA damage mimics immunosenescence and accelerates ageing

The hypothesis that increased DNA damage in the immune system drives accelerated ageing and immunosenescence is supported by a growing body of literature. A recent meta-analysis which evaluated DNA damage in the circulating immune cells of 2,403 individuals, as measured by the comet assay, reported that higher levels of damage were associated with increased mortality (Bonassi et al., 2021). Further to this correlative association, immune DNA damage has been linked causally to increased mortality and ageing. For instance, immune-specific genetic knockout of *Ercc1*, an endonuclease involved in multiple DNA repair pathways, is sufficient to phenocopy many aspects of immunosenescence and drive age-related changes in mice, including accelerated ageing of solid organs of non-immune lineages (Yousefzadeh et al., 2021b). *Ercc1*-deficient immune cells showed increased levels of the DNA damage marker γH2AX, and DNA oxidation product 8-oxo-dG, and their high expression of the cell cycle suppressor, *Cdkn2a*, indicated permanent exit from the cell cycle and senescence (Yousefzadeh et al., 2021b). Although it is not clear which haematopoietic cells drive this phenotype, the abundance of T cells in the immune system could contribute to a large extent, especially given that DNA damage accumulates in these cells and is associated with normal human ageing (discussed later). Supporting the view that T cells may be the major cell type responsible is a recent study using *Tfam* ^fl/fl^ *Cd4* ^Cre^ mice, in which the nuclear-encoded mitochondrial transcription factor A (TFAM) was knocked out in T cells only (*Tfam* encodes a protein involved in mitochondrial DNA stability, replication, and transcription) (Desdin-Mico et al., 2020). These mice had significantly shorter lifespans compared to controls with functional TFAM, and exhibited the premature onset of age-related muscular, cardiovascular, and cognitive dysfunctions, as well as age-related susceptibility to an acute viral infection, indicating premature immunosenescence (Desdin-Mico et al., 2020).

Though these studies utilise mice which harbour non-physiological mutations driving a severe pro-ageing phenotype, they suggest that DNA damage and senescence in immune cells alone is sufficient to drive age-related dysfunction. It does not exclude the possibility that DNA repair deficiency in other individual non-haematopoietic cell types may have progeroid effects. However, given that increased DNA damage in the immune system is observed during physiological ageing in T cells, and T cells are both numerous and readily available from the blood of older adults, targeting DNA repair capabilities in these cells may serve as a valuable axis in designing therapeutics to promote healthy longevity.

#### 3.1.3 Senescent T cells with DNA damage accumulate during ageing

Increased DNA damage during immunosenescence has been best characterised in the senescence of T cells during ageing. Naïve and memory T cells are long-lived cells of the adaptive immune system that become activated via their T cell receptor (TCR) to induce clonal proliferation and cytokine secretion. The specific functions of T cells are determined by their CD4^+^ or CD8^+^ subtype. Human senescent T cells are terminally differentiated, antigen experienced cells that are defined by their successive loss of expression of the co-stimulatory molecules, CD27 and CD28, together with high expression of CD57 and KLRG1 (Henson et al., 2014). T cell differentiation subsets can be determined by their relative expression of CD27 and CD45RA, thus defining four subsets: naïve (T_N_, CD27^+^CD45RA^+^), central memory (T_CM_, CD27^+^CD45RA^−^), effector memory (T_EM_, CD27^−^CD45RA^−^), and effector memory re-expressing CD45RA, known as T_EMRA_ (CD27^−^CD45RA^+^). T_EMRA_ cells are considered a senescent memory subset of T cells (Henson et al., 2014). Senescent T cells can also be identified by their high activity of senescence-associated β-galactosidase (SAβG), a classical senescence biomarker (Martinez-Zamudio et al., 2021). Thus, senescent T cells have been defined in the literature as being CD27^−^CD28^−^, CD27^−^CD45RA^+^ (T_EMRA_), or more recently as SAβG^high^ cells within the CD4^+^ or CD8^+^ subsets.

Though defined by differing markers, CD27^−^CD28^−^, T_EMRA_, and SAβG^high^ cells crucially share the characteristics both of being greatly expanded during chronological ageing and of displaying many features of senescence. The characteristics of these senescent T cell subsets are summarised in Table 1. SAβG^high^ senescent T cells accumulate in both CD4^+^ and CD8^+^ T cell compartments during ageing and are particularly enriched in the T_EMRA_ terminally-differentiated subpopulation (Martinez-Zamudio et al., 2021). Compared to non-senescent SAβG^low^ cells, senescent SAβG^high^ CD8^+^ T cells have impaired proliferative ability and high p16/INK4A expression. Furthermore, increased T cell expression of the cell cycle arrest gene p16/INK4A is an accurate predictor of chronological age in humans (Liu et al., 2009). Compared with non-senescent cells, the functions of senescent T_EMRA_ cells are severely impaired and include the inability both to stimulate proper downstream TCR signalling and to proliferate after activation - though, unlike senescent fibroblasts, their proliferative ability is not fully lost (Henson et al., 2014; Akbar et al., 2016). Senescent CD8^+^ T_EMRA_ cells are highly cytotoxic and, much like senescent fibroblasts, produce a SASP with immune-active components (namely, TNF-α and IFN-γ) after short-term activation, that may contribute to the low-grade inflammatory environment in older people (Henson et al., 2014; Henson et al., 2015; Akbar et al., 2016). T cells from older adults have lower autophagic flux (Phadwal et al., 2012), contain dysfunctional mitochondria, and have higher levels of ROS (Henson et al., 2014; Sanderson and Simon, 2017) than their younger counterparts, all of which are hallmarks of ageing (Lopez-Otin et al., 2023). It is not clear if the accumulation of T_EMRA_ (or senescent T cells) with age may be adaptive or maladaptive–for example, their hyper-cytotoxic and -secretory phenotypes may beneficially compensate for the increased burden of malignant cells during ageing, but bear the disadvantages of increased destruction of healthy self-tissues (Pereira and Akbar, 2016). Nevertheless, many of these age-related dysfunctional characteristics have been linked to the impaired function of senescent T cells, and reversing these characteristics may promote immune resilience in old people.

Senescent T cells display markers of persistent DNA damage (Table 1). They have high expression of γH2AX, low telomerase expression, and critically short telomeres (Di Mitri et al., 2011; Henson et al., 2014; Sanderson and Simon, 2017). Even though T cells retain telomerase expression for far longer than other somatic cells, telomere erosion is observed in T cells (and peripheral blood mononuclear cells, PBMCs, more generally) during ageing (Lin et al., 2015; Sanderson and Simon, 2017). Similarly, compared to non-senescent SAβG^low^ cells, SAβG^high^ senescent T cells are enriched for the DNA damage marker, 53BP1, especially at telomeres (Martinez-Zamudio et al., 2021). Further evidence for the relationship between DNA damage and ageing T cells comes from DNA repair-deficient mice (*Ercc1* ^−/Δ^), which experience an accumulation of highly-differentiated memory T cells akin to naturally aged wildtype mice (Pieren et al., 2021). Overall, senescent T cells with persistent DNA damage accumulate in older people. Due to their dysfunctional phenotype and cytotoxic nature, senescent T cells are therefore thought to contribute both to immunopathology and immunosenescence during ageing (Akbar et al., 2016; Covre et al., 2020).

### 3.2 Causes of DNA damage in T cells

**FIGURE 2:**
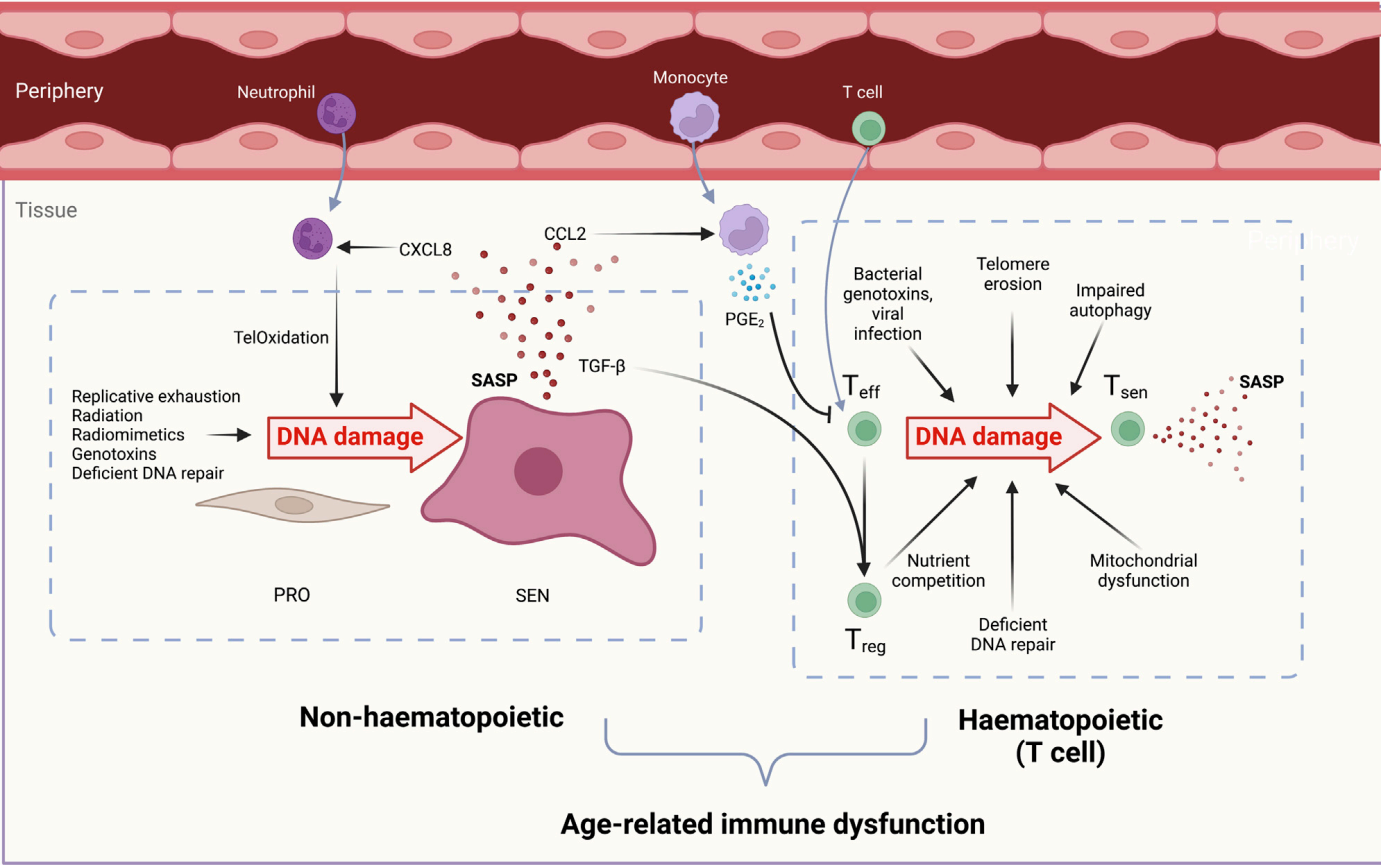
**Overview of the causes and consequences of DNA damage in both haematopoietic (T cell) and non-haematopoietic cells which contribute to immunosenescence.** DNA-damaged immune and non-haematopoietic cell senescence are linked. During ageing, senescent cells increase in both compartments due to reduced senescent cell clearance, deficient DNA repair processes, and increased genotoxin abundance. **Non-haematopoietic cells:** Proliferating cells (PRO) experience genotoxicity from radiation, excessive replication, chemotherapeutic drugs, and radiomimetics to drive persistent DNA damage, proliferation arrest, and cellular senescence (SEN). The senescence-associated secretory phenotype (SASP) contains chemoattractants such as CXCL8 that draw in peripheral neutrophils. These induce oxidative damage in stromal cell telomeric DNA, called TelOxidation, propagating further paracrine senescence. The SASP component CCL2 attracts monocytes which secrete PGE_2_ and suppress T cell functions. TGF-β in the SASP favours T_reg_ generation and a pro-suppressive, inadequate immune response during ageing. **Haematopoietic cells:** Effector T cells (T_eff_) experience genotoxic injury from bacterial genotoxins viral infections short telomeres, reactive oxygen species (ROS) from dysfunctional mitochondria, and nutrient competition with regulatory T cells (T_regs_). DNA-damaged T cells manifest senescent phentotypes in a sestrin- and ATM-dependent manner. Senescent T cells (T_sen_) are dysfunctional and contribute to inflammageing with their inflammatory SASP, tissue cytotoxicity, and immune dysfunction during ageing.

The causes of DNA damage in aged T cells may be attributed both to endogenous and exogenous sources of genotoxins, and to the endogenous decline of DNA repair factor expression. In this section we discuss the sources of DNA damage which T cells experience across the lifespan. These are summarised in Figure 2.

#### 3.2.1 Telomere attrition

Due to the highly proliferative nature of T cells upon activation, chronic stimulation over the lifecourse leads to telomere erosion (Lin et al., 2015; Sanderson and Simon, 2017). Although T cells can upregulate telomerase in order to maintain their telomeres, this ability is impaired during ageing, especially in the CD27^−^CD28^−^ senescent T cell population (Plunkett et al., 2007; Patrick et al., 2019). This leads to critically short telomeres which, as previously discussed, triggers a persistent DNA damage response and subsequent senescence. A recent study identified a novel mechanism by which human T cells may acquire telomeres delivered in extracellular vesicles from antigen-presenting cells, which increases their replicative capacity and prevents senescence (Lanna et al., 2022). It would be particularly interesting to determine whether this intercellular telomere transfer occurs as efficiently in old as it does in young individuals, as failure with ageing could potentially contribute to telomere attrition in T cells.

#### 3.2.2 Impaired autophagy and removal of dysfunctional mitochondria

Autophagy is a process by which cells degrade excess or damaged intracellular proteins and larger cargo such as organelles; altered autophagy is recognised as a hallmark of ageing (Schmauck-Medina et al., 2022; Lopez-Otin et al., 2023). Autophagic flux decreases in both B and T cells during ageing (Zhang et al., 2019; Alsaleh et al., 2020), which correlates with increased DNA damage, as measured by γH2AX expression (Phadwal et al., 2012). Autophagy is required for an adequate memory response to antigen challenge (Puleston et al., 2014). Reduced autophagy partly underlies the defective T cell response in old people: much like senescent T cells, *Atg7*-deficient T cells, which are autophagy-deficient, are impaired in their proliferative capacity after antigen exposure (Zhou et al., 2022). Ineffective autophagy in T cells from old donors also underlies other senescent T cell phenotypes that are capable of driving T cell DNA damage, such as dysfunctional mitochondrial biogenesis.

Senescent T cells from older people harbour large mitochondria with disrupted bioenergetics that release excessive reactive oxygen species (ROS) (Henson et al., 2014; Sanderson and Simon, 2017; Bharath et al., 2020). Excessive ROS release can cause oxidative damage of both genomic and mitochondrial DNA (Henson et al., 2014). This leads to a vicious cycle: since the mitochondrial genome encodes key components of the electron transport chain (ETC.), oxidative damage, combined with low levels of DNA repair operational for mtDNA, leads to further disruption in oxidative phosphorylation and hence higher ROS levels (Rong et al., 2021). ROS also contribute to telomere damage and attrition observed in T cells cultured long term *in vitro*, which can be rescued by addition of a ROS scavenger, *N*-acetyl-L-cysteine (Sanderson and Simon, 2017). Since autophagy is important in removing damaged mitochondria (termed mitophagy), the decline in basal autophagy observed in old lymphocytes is thought to contribute to accumulation of dysfunctional mitochondria that produce genotoxic ROS (Bektas et al., 2019). Accordingly, stimulating autophagy in T cells using either spermidine, metformin, or a p38 MAPK inhibitor, reduced the presence of giant dysfunctional mitochondria and restored mitochondrial bioenergetics, indicating an overall improvement in mitochondrial quality (Henson et al., 2014; Alsaleh et al., 2020; Bharath et al., 2020). It is unclear, however, whether these effects are due solely to the specific induction of mitophagy or other autophagy-induced processes, though this could be explored further - for example, with autolysosome-dependent fluorescent mitochondrial dyes such as that employed by Bektas and others (Bektas et al., 2019).

The ability of T cells to respond to DNA damage may determine their susceptibility to undergo senescence. After DNA damage, AMPK activation causes CD4^+^ T cells to increase their expression of the transcription factor, GATA3 (Callender et al., 2021). By complexing with ATR, PGC1α, and NRF2, GATA3 promotes the transcription of genes involved in mitochondrial biogenesis, thereby limiting ROS-mediated oxidative stress and preventing senescence (Callender et al., 2021). However, CD8^+^ T cells, unlike CD4^+^ T cells, do not increase GATA3 expression and mitochondrial biogenesis after DNA damage, which may partly explain their increased susceptibility to senescence and the higher proportion of senescent CD8^+^ than CD4^+^ T cells in peripheral blood (Czesnikiewicz-Guzik et al., 2008; Callender et al., 2020). This difference between CD8^+^ T cells and CD4^+^ T cells may be related to the fact that CD4^+^ T cells can develop into a variety of subsets (e.g.,.T_h_1, T_h_2, T_h_9, T_h_17, T_regs_) and mitochondrial respiration may play a role in this lineage flexibility, whereas CD8^+^ T cells are mostly of the same subtype (cytotoxic) and therefore also more limited to one type of energy production. Overall, autophagy may limit genotoxic ROS production by removing dysfunctional mitochondria and ensuring mitochondrial quality is maintained. Hence age-related decreases in autophagy may contribute to both telomere attrition and oxidative DNA damage during T cell ageing and senescence.

#### 3.2.3 Nutrient competition by regulatory T cells

While failure of mitochondrial biogenesis may be key to CD8^+^ T cell senescence, nutrient competition has been suggested to induce DNA damage and senescence in CD4^+^ T cells. Regulatory T cells (T_regs_) play a role in restricting immune activation by inhibiting immune cell proliferation to prevent pathological hyperinflammatory responses. Naïve CD4^+^ T cells, when co-cultured *in vitro* with CD25^high^FoxP3^+^ CD4^+^ T_regs_, displayed increased DNA damage markers (γH2AX and 53BP1) and active DDR signalling (p-ATM and p-Chk2) as well as other senescence phenotypes, including cell cycle arrest and high senescence-associated β-galactosidase activity (Ye et al., 2012; Liu et al., 2018). Since co-culturing in high glucose prevented these DNA damage phenotypes, this indicates that insufficient glucose, potentially due to the high consumption by T_regs_, may be involved. Indeed, in naïve T cells co-cultured with T_regs_, the resulting low glucose levels and subsequent p-AMPK activation may stimulate mitochondrial biogenesis and a shift from glycolysis to oxidative phosphorylation, driving ROS production and DNA damage (as discussed earlier)*.* T_reg_-mediated immune regulation in this way could plausibly be a short-term advantageous mechanism, as induction of senescence in the effector T cells would limit their functions and therefore an overexuberant immune response. However, as senescent T cells display an inflammatory SASP and poor proliferative ability, this regulation may have longer-term drawbacks by impairing immune function. Furthermore, this process may be exacerbated in an ageing context where tissues are enriched with T_regs_ (Agius et al., 2009), which could plausibly lead to excessive induction of senescence in effector T cells.

#### 3.2.4 Exposure to infectious agents

Infections can directly and indirectly induce DNA damage and cellular senescence. Since immune cells accumulate at sites of infection, these cell types are ‘first responders’ which are repeatedly exposed to pathogen-derived genotoxic insults. Bacterial genotoxins, such as the typhoid toxin from *Salmonella* Typhi induces a γH2AX response induced by a genotoxin, or RING, in human fibrosarcoma cells, which is dependent on ATR-mediated hyperphosphorylation of RPA (Ibler et al., 2019). These RINGs comprise a non-canonical and persistent DDR that induces cellular senescence. Toxin-induced senescent cells have a unique SASP (txSASP) that further drives paracrine senescence and promotes *Salmonella* infection (Ibler et al., 2019). Overall, bacterial genotoxins may have evolved to enhance bacterial infection and transmissibility due to their pro-senescence effects.

Like some bacteria, virus infection is also an inducer of DNA damage and senescence (Lee et al., 2021; Gioia et al., 2023). Virus-induced senescence (VIS) may have evolved as an innate mechanism to inhibit viral exploitation of host replication machinery. However, some viruses evolved to exploit their pro-senescence effects to increase their infection ability. For example, coronavirus (e.g., SARS-CoV2, which causes COVID-19) induces cellular senescence and the SASP; this virus-induced SASP promotes upregulation of viral entry proteins, ACE2 and TMPRSS2, and downregulation of antiviral factors in adjacent cells, thus enhancing further infection susceptibility (Camell et al., 2021; Lee et al., 2021). SARS-CoV2 infection causes DNA damage *in vitro* and *in vivo* in COVID-19 patients: distinct SARS-CoV2 viral proteins both promote degradation of the DDR enzyme, Chk1, and outcompete 53BP1 for binding to DNA damage-induced long non-coding RNAs, thus preventing repair of DSBs (Gioia et al., 2023). Importantly, removing senescent cells improved the survival of mice after beta-coronavirus infection, indicating the core role of VIS in the pathology of viral infection (Camell et al., 2021; Cox and Lord, 2021).

Since the immune system is intimately involved with infection resolution, it is likely that immune cells are frequently exposed to bacterial genotoxins and viral senescence inducers. Indeed, exposure of activated T cells to cytolethal distending toxin, a genotoxin produced by many pathogenic Gram-negative bacteria, induces DNA damage, senescence, and ATM-induced SASP (Mathiasen et al., 2021). Suppression of effector T cell function by inducing senescence may be an evolved strategy by pathogens to suppress the adaptive immune response (Mathiasen et al., 2021). Other pathogens, such as the parasites belonging to the *Leishmania* genus, have exploited this to induce senescent T cells with DNA damage, in order to hijack their hyper-cytotoxic and hyper-secretory functions and further drive immunopathology (Covre et al., 2018; Covre et al., 2020). Furthermore, IFN-α, as part of the antiviral Type I interferon response, may induce senescent phenotypes in T cells by inhibiting telomerase and driving telomere erosion (Reed et al., 2004; Lanna et al., 2013). Therefore, infections may drive immune DNA damage by both direct effects of the infectious agent, and by secondary effects of cytokine release on immune cell biology.

#### 3.2.5 Chronic activation and reduced DNA repair factor expression

Heightened inflammation with increasing age, termed ‘inflammageing’, is now recognised as a major hallmark of ageing (Schmauck-Medina et al., 2022), and has been heavily implicated in driving immunosenescent phenotypes. Chronic activation of the immune system, such as in chronic viral infections and autoimmune diseases (e.g., rheumatoid arthritis), fosters a highly inflammatory environment. These disease conditions feature many of the ageing hallmarks, and have therefore been proposed to be conditions of accelerated ageing (Alsaleh et al., 2022). These conditions also mimic the accumulation of senescent CD28^−^ T cells observed during normal ageing, and so they may provide valuable contexts to understand immunosenescence (Alsaleh et al., 2022).

T cells from donors with a chronic virus infection (e.g., HBV, HCV or HIV) have short telomeres and display high levels of DNA damage (Ji et al., 2019); indeed, the severity of liver fibrosis negatively correlates with T cell telomere length in chronic HCV infection (Hoare et al., 2010; Hohensinner et al., 2011). T cells from chronically virus-infected donors are hypersensitive to DNA damage agents, such as topoisomerase I (TOP1) inhibition by camptothecin (Ji et al., 2019). The decrease in T cell genome stability in these patients can be attributed to 1) chronic activation-induced proliferation leading to telomere erosion and 2) decreased expression and activity of DNA repair factors, such as TOP1, ATM, and the telomere shelterin complex component, TRF2 (Nguyen et al., 2018; Zhao et al., 2018; Ji et al., 2019). Importantly, restoring expression of the DDR regulator, ATM, was able to rescue IL-2 production by the T cells of patients with chronic HCV infection (Zhao et al., 2018).

Patients with autoimmune diseases, such as rheumatoid arthritis (RA), display an expansion of senescent T cells with a high burden of DNA damage (Shao et al., 2009; Hohensinner et al., 2011). These cells display critically short telomeres, which may be related to impaired upregulation of telomerase upon activation in naïve CD4^+^ T cells (Fujii et al., 2009). Furthermore, CD4^+^ T cells from RA patients display an increased burden of DNA damage, both at baseline and in response to ionising radiation (Shao et al., 2009). As with chronic HCV infection, the genome instability in T cells from RA patients is partly attributed to insufficiency of the DDR enzyme, ATM, and can be rescued by ectopic ATM overexpression (Shao et al., 2009). Furthermore, senescent CD4^+^ T cells from RA patients are deficient in their expression of the DNA repair nuclease, MRE11A, leading to their increased telomeric damage and ability to injure tissues (Li et al., 2016).

Thus, in the contexts of chronic immune activation where accelerated immune ageing is observed, genome instability arises because of insufficiency in key DNA repair pathway enzymes. It is unclear why DNA repair factor expression decreases in these conditions, and whether boosting DNA repair processes may be therapeutically advantageous. It remains to be assessed whether the phenotype of reduced DNA repair factor expression is present in normal human immune system ageing.

### 3.3 DNA damage drives maladaptive senescent T cell phenotypes

#### 3.3.1 ATM drives sestrin 2-dependent MAPK activation

T cells experience DNA damage across the lifespan. Chronic DNA damage leads to persistent DDR signalling but without completion of DNA repair. This sustained DDR experienced by senescent T cells contributes to their maladaptive phenotypes. Activation of ATM during the DDR drives activation of metabolic master regulator AMPK, which complexes with Sestrin 2, a stress-response protein, to activate several downstream MAPKs (ERK, JNK and p38) (Lanna et al., 2017). Activation of these individual MAPKs induces distinct senescent phenotypes, such as impaired proliferation, telomerase insufficiency, hypersecretion, low autophagic flux, and high ROS (Lanna et al., 2017). Knockdown of Sestrin 2 or pharmacological inhibition of MAPKs rejuvenated senescent T cell phenotypes and, interestingly, reduced γH2AX expression (Henson et al., 2014; Lanna et al., 2017). This indicates a two-way relationship between DNA damage-ATM activation, and AMPK-Sestrin 2-MAPK activation, potentially mediated by the effects of reducing ROS levels through MAPK inhibition (Lanna et al., 2017).

#### 3.3.2 Gain-of-function senescent phenotypes

DNA damage and ATM activation in senescent T cells elicits gain-of-function characteristics, via Sestrin 2, that may drive immunopathology in inflammatory contexts, such as ageing and autoimmune diseases. Senescent CD8^+^ T cells acquire an NK cell-like phenotype (Strauss-Albee et al., 2014), upregulating a range of NK receptors (NKRs) such as the activating receptor NKG2D, which is dependent on the expression of Sestrin 2 (Pereira et al., 2020). Senescent NKG2D^+^ T cells, displaying defective TCR-dependent signalling, are capable of an alternative NKG2D-mediated activation to elicit their cytotoxic functions (Pereira et al., 2020). This process could feasibly have evolved to compensate for the declining canonical T cell activation response in ageing. However, in the context of autoimmune disease, it has been suggested that NKR-activated T cells may drive the destruction of self-tissues in which cells upregulate NK ligands due to the high-stress inflammatory environment (Covre et al., 2020; Pereira et al., 2020). This has been hypothesised to drive immune-mediated tissue damage, implicating a causal role of senescent T cells in driving immunopathology. Hence DNA damage and ATM activation in senescent T cells can drive both loss-of-function (e.g., poor proliferation) and gain-of-function (e.g., NKR upregulation) phenotypes, both of which contribute to poor antigen-specific immunity and immunopathology during ageing.

### 3.4 Non-haematopoietic cell senescence promotes immunosenescence

Immunosenescence is also driven by the increasingly inflammatory niche of older tissues, due to the increased burden of senescent non-haematopoietic ‘stromal’ cells (Chambers et al., 2021a). Since persistent DNA damage is a major driver of senescence, as previously discussed, efforts to promote DNA repair may attenuate senescent cell burden and the inflammatory tissue environment, with the potential to rejuvenate immune responses during ageing.

#### 3.4.1 Mechanisms of senescent cell accumulation during ageing

Immunosenescence contributes to the accumulation of senescent stromal cell during ageing. In healthy young individuals, turnover of senescent cells is homeostatically regulated: senescent cells are generated (for example, acutely at sites of wounds (Demaria et al., 2014)) and then killed by immune cells such as CD8^+^ T cells and Natural Killer (NK) cells, and cleared by macrophages through phagocytosis (Oishi and Manabe, 2016; Pereira et al., 2019). However, senescent cells accumulate during ageing and cause age-related disease pathology. This accumulation is driven both by an increase in stressors that generate senescent cells, and by impaired immunosurveillance and reduced clearance of senescent cells during ageing, partly due to immunosenescence (Ovadya et al., 2018; Pereira et al., 2019). For example, senescent cells increase their expression of immune evasion molecules such as HLA-E, thus inhibiting their clearance by engaging the immunosuppressive T cell NKG2A receptor, an NKR which is upregulated upon T cell senescence (Pereira et al., 2019; Pereira et al., 2020). Similarly, senescent cell PDL-1 expression prevents clearance by engaging the PD-1 receptor on CD8^+^ T cells (Wang et al., 2022). Furthermore, the cytotoxicity of NK cells decreases in older people, with the cells acquiring defects in their ability to deliver cytotoxic granules to the immune synapse (Hazeldine et al., 2012), which may impair their ability to kill senescent cells. The ability of macrophages to undergo phagocytosis also becomes defective during ageing, at least in mice (Solana et al., 2012; Stranks et al., 2015). Indeed, strategies that remobilise the immune system to target senescent cell (or ‘seno’-) antigens using CAR T cells (Amor et al., 2020), seno-directed vaccines (Suda et al., 2021), and immune checkpoint blockade (Wang et al., 2022) have been successful in attenuating senescent cell burden and promoting health in ageing mice.

The aged immune system is also capable of inducing senescence of non-haematopoietic cells. In the *Vav-iCre* ^+/−^;*Ercc1* ^-/fl^ immunosenescence model, DNA damage in haematopoietic cells alone led to accelerated ageing and increased senescent cell burden in non-haematopoietic tissues, indicating a potential role of bystander/paracrine senescence between the different cell types (Yousefzadeh et al., 2021b). Immune cells also perpetuate tissue senescence by inducing DNA damage. Senescent liver cells secrete IL-8/CXCL8 within their SASP, which recruits neutrophils that release ROS that in turn damage the telomeres of bystander hepatocytes in a process termed TelOxidation (Lagnado et al., 2021). Indeed, TelOxidation may be exacerbated during ageing through non-directional tissue infiltration by neutrophils with impaired chemotaxis, which can cause widespread collateral damage and bystander senescence (Sapey et al., 2014). Therefore, DNA damage of cells both within and outside of the immune system contributes to the increased senescent cell burden and subsequent inflammatory niche, which is capable of impairing immunity during ageing.

#### 3.4.2 SASP-mediated suppression of antigen-specific immunity

Senescent cells compromise immunity in an aged context. The primary mechanism by which this occurs is through the secretion of components of the SASP. For example, the cytokine CCL2/MCP-1, produced by senescent fibroblasts in culture and the skin of old people, is an inflammatory monocyte chemoattractant (Pereira et al., 2019; Chambers et al., 2021a). Monocytes are recruited to the skin at sites of cutaneous immune challenge in old people, and through their release of PGE_2_, suppress the adaptive proliferative responses of local resident T cells (Chambers et al., 2021a). SASP components also impact the differentiation of effector T cells recruited during an immune response and promote an excessively suppressive environment. This may account for the observation that old mice and people exhibit poor clearance of influenza infection compared to younger individuals (Thompson et al., 2004; Lorenzo et al., 2022). TGF-β, a component of the SASP, can promote the differentiation of FoxP3^+^ regulatory T cells (T_regs_). There are more T_regs_ in the lungs of old mice than young mice after influenza infection, and this can be attenuated by mAb-mediated blockade of TGF-β, supporting the importance of TGF-β in immune suppression (Lorenzo et al., 2022). To determine the role of senescent cells in dampening this influenza-specific immunity, old mice were treated prior to infection with dasatinib and quercetin (D + Q), a senolytic combination that inhibits the pro-survival pathways that are hyperactivated in senescent cells. Clearance of senescent cells in this way reduced both TGF-β levels and FoxP3^+^ T_reg_ accumulation, and improved the response to influenza infection, suggesting that senescent cells were responsible for suppressing adequate immune responses during lung infection in old mice (Lorenzo et al., 2022). In younger life, this pathway may be advantageous in restricting an overexuberant response in the lung. However, an over-suppressive immune environment generated by accumulation of T_regs_ may contribute to the impaired clearance of influenza infection in an aged context. Furthermore, senescent cells and their SASP drive mortality during coronavirus infection: indeed, *in vivo* senolysis of coronavirus-induced senescent cells, by the senolytics D + Q or navitoclax, reduced peripheral SASP levels, COVID-19-like immunopathology, and mortality in animal models of the infection (Camell et al., 2021; Lee et al., 2021; Pastor-Fernandez et al., 2023). It would be interesting to investigate whether the senolytic removal of certain cell types, such as those either of immune or non-immune origin, would be sufficient to limit the SASP and senescence-related pathology in these diseases.

## 4 “Genoprotection” in interventions targeting immunosenescence

Under our hypothesis that immunosenescence is caused by DNA damage, it follows that interventions that inhibit DNA damage and promote DNA repair should attenuate immunosenescence. However, in the absence of known DNA damage inhibitors, this has not been tested. Nevertheless, a variety of treatment interventions have been reported to restore immunity in older people. Interestingly, the efficacy of many of these can be partly explained by their ability to promote genome stability - such interventions therefore may be described as “genoprotective.” This serves as indirect evidence that inhibiting DNA damage and promoting DNA repair may improve immune resilience in old people. This section will consider some of these potential immunoprotective treatments (spermidine, metformin, mTOR inhibitors, p38 MAPK inhibitors, and vitamin D_3_) and offer a genoprotective mechanism of action that may contribute to their success. We further discuss the potential merit in exploring nicotinamide riboside (NR) or enoxacin supplementation as interventions to target immunosenescence, given the evidence for their genoprotective abilities in limiting DNA damage. These interventions, along with their relevant effective *in vitro* and *in vivo* doses, are summarised in Table 2.

**TABLE 2:** Summary of immunosenescence interventions that may genoprotective.

Intervention	Evidence for beneficial effect on immunosenescence	Proposed mechanism(s) of action	Evidence for drug genoprotection	Effective dose	
				*In vitro*	*In vivo* (human)
Spermidine	Improved vaccine-induced HCV- and RSV- specific responses in CD8^+^ T cells from old people Alsaleh et al. (2020)	Enhances hypusination of eIF5A and translation of TFEB, leading to induction of autophagy-related gene expression Zhang et al. (2019)	Promoted DSB repair by homologous recombination in U-2 OS cells Lee et al. (2019)	0.01–0.5 mM Puleston et al. (2014), Lee et al. (2019), Alsaleh et al. (2020)	1.2 mg/day Schwarz et al. (2018), though higher doses (15 mg/day) can be tolerated Senekowitsch et al. (2023)
	Improved flu-specific immune memory in old mice Puleston et al. (2014)	Increases autophagy in B cells and T cells from old individuals Zhang et al. (2019), Alsaleh et al. (2020)			
Metformin	Improved survival (in women) from COVID-19 (Bramante et al., 2021) and other studies, reviewed in Justice et al. (2021)	Multiple targets, many of which act through AMPK activation Kulkarni et al. (2020)	Elicited pseudo-DDR in cancer cells Vazquez-Martin et al. (2011)	5 mM Vazquez-Martin et al. (2011), Lee et al. (2016)	500 mg twice daily, then increased to 2,000 mg daily at the end of 2 weeks led to average plasma concentration 18 μM metformin Kulkarni et al. (2018)
			Decreased %γH2AX^+^ and %8-oxo-dG^+^ intestinal stem cells in aged or paraquat-treated *Drosophila* Na et al. (2013)		
mTOR inhibition	6-week pre-treatment with RAD001 vs. placebo prior to influenza vaccination increased antibody titre and decreased %PD-1^+^ T cells Mannick et al. (2014)	Decreases %PD-1^+^ T cells Mannick et al. (2014)	Attenuated immunosenescence effects of immune cell DNA damage in *Vav-iCre* ^*+/−*^ *;Ercc1* ^*-/fl*^ mice Yousefzadeh et al. (2021b)	0.01–1 µM Saha et al. (2014), Dominick et al. (2017), Yang et al. (2022)	Rapalogues RTB101 at 10 mg/day or combined RTB101 10 mg/day + RAD001 0.1 mg/day both beneficial Mannick et al. (2018)
			Dietary restriction (which restricts mTOR activation) prolonged life in DNA repairdeficient mice Vermeij et al. (2016)		
	6-week dual BEZ235/RAD001 vs*.* placebo treatment in old people increased influenza antibody titre and decreased respiratory tract infections 1 year later Mannick et al. (2018)	Increases type I interferon antiviral gene expression Mannick et al. (2018)	Rapamycin treatment decreased # 53BP1 foci in WRN-deficient fibroblasts Saha et al. (2014)		1 mg/day rapamycin in older adults (average peak blood rapamycin concentration 7.88 nM (range 5.14–12.91)) Kraig et al. (2018)
			Rapamycin increased post-translational expression of DNA repair enzymes NDRG1 and MGMT Dominick et al. (2017)		
p38 MAPK inhibitors	Improved response to VZV challenge in skin in old people Vukmanovic-Stejic et al. (2018), Chambers et al. (2021a)	Improves efferocytosis of macrophages promoting immune resolution after blister challenge De Maeyer et al. (2020)	Restored telomerase expression and reduced reactive oxygen species in senescent T cells Henson et al. (2014)	3 µM losmapimod De Maeyer et al. (2020), Chambers et al. (2021a)	15 mg twice daily losmapimod De Maeyer et al. (2020), Chambers et al. (2021a)
				500 nM BIRB 796 Henson et al. (2014)	
		Decreases CCL2 expression from skin to decrease inflammatory monocyte recruitment to allow adaptive T cell response Chambers et al. (2021a)	Restored young morphology and proliferation in senescent DNA repair-deficient Werner syndrome fibroblasts Davis et al. (2005)	10 µM SB203580 Davis et al. (2005)	
Vitamin D_3_	14-week vitamin D_3_ supplementation improved cutaneous response to VZV challenge in old people Chambers et al. (2021b)	Decreases inflammatory monocyte recruitment to site of challenge and allowed resident memory T cell adaptive response Chambers et al. (2021b)	1,25(OH)_2_ treatment increased recruitment of DNA repair factors to DNA lesions after ionising radiation in human fibroblasts Graziano et al. (2016)	100 nM Graziano et al. (2016), Elkafas et al. (2020)	6400 IU of vitamin D_3_ per day for 14 weeks; average serum concentration of 25(OH)D after supplementation was ∼125 nM
			Vitamin D_3_ upregulated DNA sensors and effectors and diminished DNA damage in myometrial stem cells isolated from rats developmentally treated with diethylstilbestrol Elkafas et al. (2020)		
Nicotinamide riboside	Not yet assessed	Restores NAD^+^ levels that naturally decline with age Fang et al. (2019)	NR enhanced SIRT1-mediated deacetylation of Ku70 in the DNA-PK complex to promote NHEJ-mediated repair of DSBs in ATM-insufficient human neuroblastoma cells Fang et al. (2016)	0.5–1 mM NR Fang et al. (2016), Fang et al. (2019)	500 mg of NR twice daily for 6 weeks increased PBMC concentration of NAD^+^ by 60% compared to placebo; median NAD^+^ levels from 7.7 to 12.2 pmol/mg protein Martens et al. (2018) 1 g/day NR for 21 days increased blood NAD^+^ by > 2-fold (47.75 μM supplementation *versus* placebo 20.90 μM) Elhassan et al. (2019)
		Activates SIRT1 to promote mitophagy Fang et al. (2019)	NR promoted RAD51-mediated HR repair of DSBs Fang et al. (2019) NR supplementation in aged mice decreased %γH2AX^+^ muscle stem cells Zhang et al. (2016)		
Enoxacin	Not yet assessed	Increases activity of RNAi machinery, DICER; promotes the generation of non-coding RNAs recruited to sites of DNA damage (DDRNAs) Gioia et al. (2019)	Treatment of HeLa cells prior to ionising radiation increased accumulation of 53BP1 foci at DNA lesions and decreased tail moment in neutral comet assay (readout for DSBs) Gioia et al. (2019)	50 µM Gioia et al. (2019)	400 mg twice daily enoxacin increased average plasma drug concentrations of 15.86 µM (SD 3.53, range 10.26–24.06 µM) Hamel et al. (2000)
				*C. elegans* lifespan extension when grown on plates supplemented with 100 μg/mL (312.19 µM) enoxacin Pinto et al. (2018)	

### 4.1 Known interventions against immunosenescence are “genoprotective”

#### 4.1.1 Spermidine

Spermidine is a naturally-derived polyamine that has been shown to improve antigen-specific B and T cell responses in cells from old donors, through increasing autophagy (Zhang et al., 2019; Alsaleh et al., 2020). Spermidine is used as a substrate for the post-translational hypusination of eIF5A, a factor that enhances the translation of otherwise hard-to-translate poly-proline sequences. One such poly-proline-containing protein is TFEB, a transcription factor that drives the expression of autophagy genes. Thus, spermidine treatment increases autophagy in both old B and T cells through increased production of TFEB (Zhang et al., 2019; Alsaleh et al., 2020). As discussed, dysfunctional mitochondria accumulate in T cells from old people, and these produce ROS that damage the DNA and promote telomere attrition (Sanderson and Simon, 2017). Promoting autophagy by a different drug, metformin, has been shown to restore mitochondrial function in CD4^+^ T cells in old people (Bharath et al., 2020); by extension the autophagy-activating effect of spermidine may also restore mitophagy and mitochondrial health, thus protecting the DNA from ROS-mediated damage. Rejuvenating autophagy by spermidine may therefore be considered as an intervention that protects the genome, i.e., that is genoprotective.

In addition to their action through autophagy, polyamines such as spermidine may directly limit DNA damage and promote DNA repair. For example, spermine (spermidine’s derivative) reduces the presence of DNA oxidation products after damage by H_2_O_2_ (Muscari et al., 1995). Spermidine was also found to promote homologous recombination repair (HRR) mediated by RAD51 after DNA damage (Lee et al., 2019). Consistent with a core role for polyamines in DNA repair, inhibition of the polyamine biosynthesis pathway using DFMO (which inhibits ornithine decarboxylase) prolonged the presence of DNA damage after ionising radiation or PARP inhibition by olaparib (Lee et al., 2019). Though several polyamines improved HRR after DNA damage, spermine was found to be most efficient (Lee et al., 2019). Spermidine and other related polyamines may therefore elicit genoprotective effects and alleviate immunosenescence by acting as antioxidants, promoting repair, and reducing ROS burden through increased autophagy. Spermidine supplementation in humans is tolerated at doses tested up to 15 mg/day, though we note that supplementation does not detectably change blood spermidine levels, despite eliciting its beneficial anti-ageing effects (Schwarz et al., 2018; Wirth et al., 2018; Senekowitsch et al., 2023). Current clinical trials investigating whether spermidine supplementation (e.g., NCT05421546, spermidine at 6 mg/day) improves vaccine responses in old people will be greatly informative in translating *in vitro* studies to population interventions.

#### 4.1.2 Metformin

The anti-diabetic drug metformin is a potential geroprotector affecting a number of the hallmarks of ageing (Kulkarni et al., 2020); it may also improve immune resilience in older people (Justice et al., 2021). Metformin has polypharmacological effects, one of which we propose is genoprotective. Metformin is an AMPK activator and thus induces autophagy via activating phosphorylation of ULK1 (Kulkarni et al., 2020). In this way it can induce the removal of dysfunctional mitochondria (sources of genotoxic ROS) to prevent DNA damage. Indeed, metformin treatment of T cells from older people restored their autophagy and mitochondrial bioenergetics.

Metformin can affect the DNA damage response by driving a pseudo-DDR that protects genome stability. Epidermoid carcinoma cells treated for 2 days with metformin accumulated γH2AX foci and high levels of the phosphorylated DDR orchestrator, p-ATM (Ser ^1981^), in the absence of any detectable DNA lesions (Vazquez-Martin et al., 2011). Furthermore, metformin treatment of A549 lung cancer cells modulated the expression of p-Chk2, p53 and p53R2 (a p53 target gene) after UVC-induced DNA damage (Lee et al., 2016). Importantly, supplementation of metformin in *Drosophila* diminished the proportion of γH2AX^+^ and 8-oxo-dG^+^ intestinal stem cells undergoing acute paraquat-induced oxidative stress or chronological ageing, indicating the genoprotective effects of metformin (Na et al., 2013). Though some *in vitro* studies have used high metformin concentrations (5 mM) that would not be attainable *in vivo*, it is encouraging that a well-tolerated treatment regimen in older people was associated with upregulated expression of several DNA repair pathways in muscle biopsies (base excision repair, mismatch repair, and BRCA-mediated repair) (Kulkarni et al., 2018). Metformin may therefore ensure DNA stability by limiting damage by oxidative stress and further kickstarting a DDR and DNA repair.

#### 4.1.3 mTOR inhibitors

Mammalian Target of Rapamycin (mTOR) is a master regulator kinase involved in nutrient sensing and cellular anabolic activity. mTOR inhibition is a potent life extension strategy in mammals (Harrison et al., 2009) and may have therapeutic advantages in many age-related disease contexts, including immunosenescence (Walters and Cox, 2018; Cox et al., 2020). At high doses, mTOR inhibitors such as rapamycin are immunosuppressive. However, low-dose rapamycin is immunoprotective; it promoted survival against lethal influenza infection in mice (Keating et al., 2013), and other acute pathogenic insults as reported in a recent meta-analysis of 23 studies in mice (Phillips and Simons, 2022). Low-dose mTOR inhibition strongly attenuates immunosenescence, as demonstrated by clinical trials in old people: mTOR inhibition by either RAD001 monotherapy, or dual RAD001/BEZ235 therapy, improved influenza vaccination responses and decreased subsequent respiratory tract infection rates 1 year after treatment (Mannick et al., 2014; Mannick et al., 2018), while treatment with BEZ235 (also named RTB101) showed trends towards immune improvement (Mannick et al., 2021). Moreover, the low nanomolar doses used in *in vitro* studies are achieved *in vivo* [e.g., in one study, 1 mg/day of rapamycin raised the average blood concentrations of the drug to 7.88 nM (range 5.14–12.91) (Kraig et al., 2018)]. The mechanism for immunoprotection by mTOR inhibition is not fully understood, but it has been observed to decrease %PD-1^+^ T cells, increase B cell antibody titres, and increase type I interferon gene expression in peripheral blood (Mannick et al., 2014; Mannick et al., 2018; Mannick et al., 2021).

mTOR inhibition modulates senescence and may promote genome stability. In the *Vav-iCre* ^*+/−*^ *;Ercc1* ^*-/fl*^ DNA damage immunosenescence model, rapamycin improved the immune response to a model antigen, and reduced the expression of the cell cycle inhibitors, *CDKN2A* and *CDKN1A* in T cells and several other tissues, though the effect of rapamycin treatment on markers of DNA damage in the immune cells was not assessed (Yousefzadeh et al., 2021b). Direct evidence that mTOR inhibition is genoprotective comes from the observation that long-term culture with rapamycin reduces the number of 53BP1^+^ DNA damage foci in fibroblasts deficient in the DNA repair factor, WRN (Saha et al., 2014). In addition to this, dietary restriction (DR), a well-known life extension strategy that converges on the inhibition of the mTOR signalling pathway (Cox and Mattison, 2009), elicited a 3-fold increase in median lifespan in both DNA repair-deficient *Ercc1* ^*Δ/−*^ and *Xpg−/−* progeroid mice compared to *ad libitum*-fed mutant mice (Vermeij et al., 2016). Importantly, genome damage, as measured by the fraction of neuronal cells showing γH2AX staining, was reduced in DR-fed mice, suggesting that DR could alleviate genome instability in the context of DNA repair deficiency (Vermeij et al., 2016). Since the mTOR pathway is inhibited during nutrient deprivation (particularly via reduced phosphorylation of one of its downstream targets, p-Akt (Ser^473^), this indirectly suggests that DR-induced genome stability may be through mTOR inhibition (Vermeij et al., 2016). However, since neither rapamycin treatment nor genetic inhibition of mTOR could fully recapitulate the DR-induced life extension in *Ercc1* ^*Δ/−*^ mice, the pro-longevity effects of DR may act through additional mechanisms independent of mTOR (Birkisdóttir et al., 2021).

The mechanism by which mTOR inhibition may promote genome stability is not fully understood. As mTOR inhibits ULK1 and autophagy, mTOR suppression and subsequent induction of autophagy may be genoprotective by removing intracellular sources of genotoxicity, such as the damaged mitochondria that produce high levels of ROS. mTOR may also influence DDR pathways. Several strains of long-lived mice show decreased mTOR activity in a number of tissues (Dominick et al., 2015), associated with high levels of the DNA repair factors MGMT and NDRG1 (Dominick et al., 2017). This is recapitulated by mTOR inhibition both *in vitro* and *in vivo*: mTOR inhibition by rapamycin treatment led to increased protein expression of MGMT and NDRG1 via a post-transcriptional mechanism through modulation of the CCR4-NOT complex (Dominick et al., 2017). Increased protein expression of these DNA repair factors may therefore promote DNA repair of existing damage, thus attenuating senescent phenotypes. Interestingly, in the case of CD8^+^ T cells where mTOR activity is low, rapamycin treatment may have beneficial effects that are independent of mTOR (Henson, 2015). These mTOR-independent mechanisms remain to be explored.

#### 4.1.4 p38 MAPK inhibitors

MAPK signalling occurs in response to growth factors, leading to cell proliferation; alternatively, members of the MAPK family are implicated in stress responses, including following DNA damage (Yacoub et al., 2001). Consistent with this, and the high levels of DNA damage in senescence, p38 MAPK is more active in both senescent T cells and non-haematopoietic cells compared to non-senescent counterparts (Lanna et al., 2014; Davis et al., 2016; Callender et al., 2018). p38 MAPK inhibitors also successfully dampen the inflammatory SASP of senescent cells, partly through inhibiting p38-DDR signalling (Alimbetov et al., 2016). p38 MAPK inhibitors, such as losmapimod and BIRB796, improve both senescent phenotypes and responses to immune challenge in older people (Vukmanovic-Stejic et al., 2018; Chambers et al., 2021a). p38 MAPK inhibition increases autophagy and telomerase expression, and reduces levels of intracellular ROS in senescent T cells (Henson et al., 2014). Thus, by restoring T cell telomere length and removing genotoxic ROS, p38 MAPK inhibition is a genoprotective strategy. Further evidence for the genoprotective effects of p38 MAPK inhibition is evidenced by the effects of SB203580 treatment, which rejuvenates both the senescent morphology and proliferative potential of DNA repair-deficient Werner Syndrome fibroblasts (Davis et al., 2005), although this treatment may have off-target effects (Lali et al., 2000). Furthermore, long-term (>3 months) treatment with current p38 MAPK inhibitors (such as losmapimod) is unfavourable due to hepatotoxicity, though short-term treatment prior to immune challenge may be beneficial. Although current p38 MAPK inhibitors are limited by their low therapeutic index and specificity, drugs with higher specificity and selectivity for MAPK isoforms are in development (Davis et al., 2016).

#### 4.1.5 Vitamin D_3_

Vitamin D_3_ supplementation has been shown to improve cutaneous immune responses in old people, in part due to the effects of reducing inflammatory monocyte recruitment to the site of antigen challenge (Chambers et al., 2021b). Interestingly, vitamin D_3_ modulates DNA repair factor activity and cellular senescence (Graziano et al., 2016). For example, fibroblasts that have undergone oncogene-induced senescence (OIS) fail to repair DNA damage due to insufficient expression of BRCA1 and the vitamin D receptor (VDR); VDR knockdown recapitulated both BRCA1 insufficiency and the senescence phenotypes observed in OIS (Graziano et al., 2016). Intriguingly, treatment of OIS fibroblasts with active vitamin D_3_ (1,25(OH)_2_-D_3_) enhanced the recruitment of DNA repair factors to DNA lesions after ionising radiation (Graziano et al., 2016). This reveals an axis connecting vitamin D_3_, DNA repair factor activity, and senescence. Furthermore, vitamin D_3_ treatment reduced DNA damage foci in myometrial stem cells isolated from rats developmentally exposed to diethylstilbestrol (DES) (Elkafas et al., 2020). Indeed, *in vitro* treatment with vitamin D_3_ enhanced expression of both DNA damage sensors (in the MRN complex) and DDR effector proteins (RAD51 and BRCA2) after DES treatment (Elkafas et al., 2020). Additionally, vitamin D_3_ antagonised the effects of DNA-damaging treatment by upregulating MRE11 and other DDR genes (Elkafas et al., 2020). These data suggest that vitamin D_3_ is genoprotective and its supplementation in humans could improve immune responses in old people by limiting DNA damage. This vitamin D_3_-DNA repair axis deficiency may be particularly pertinent in older adults, where vitamin D deficiency is common. Importantly, supplementation in humans elevates vitamin D_3_ blood to concentrations similar to those used in *in vitro* studies that demonstrate improved DNA repair (Table 2); however, the effect of supplementation on DNA damage readouts in either haematopoietic or non-haematopoietic cells in people remains to be explored.

### 4.2 Novel genoprotective interventions for immunosenescence

#### 4.2.1 Nicotinamide riboside supplementation

Energy homeostasis is critical for correct immune function, yet immune energetics have remained largely ignored within the extensive body of research on NAD^+^. NAD^+^ levels decline during normal ageing and this contributes both to increased DNA damage and mitochondrial dysfunction, both processes discussed in this review to be drivers of immunosenescence (Yoshino et al., 2018). The age-related decline in NAD^+^ is due, in part, to the increase in its consumption by NADases, such as CD38, which are upregulated in tissue-resident macrophages in response to the SASP (Chini et al., 2020; Covarrubias et al., 2020). Additionally, NADases, such as DNA damage-induced poly-ADP ribosyl polymerases (PARPs), become hyperactivated in senescence due to the increase in DNA lesion burden, leading to NAD^+^ decline (Fang et al., 2014; Yoshino et al., 2018). The overactivation of these NAD-consuming enzymes restricts the activity of other pro-longevity NAD-dependent enzymes, the sirtuins: e.g., SIRT1, an NAD-dependent deacetylase that regulates mitochondrial biogenesis by deacetylating PGC-1α (Fang et al., 2016). Restoring SIRT1 activity by NR supplementation increases mitophagy, removal of ROS-producing dysfunctional mitochondria, and lifespan in progeroid worms mutant for the DNA repair factor, *wrn* (*wrn-1(gk99)*), and in progeroid mice that are deficient in the DDR master regulator, *Atm* (Fang et al., 2016; Fang et al., 2019). Indeed, in progeroid *Tfam* ^*fl*/fl^ *Cd4* ^Cre^ mice that accumulate senescent T cells with dysfunctional mitochondria, NR supplementation reversed tissue inflammation and senescence, although the explicit effect of this treatment on immune responses was not reported (Desdin-Mico et al., 2020). NR supplementation also attenuated inflammageing in *Atm* ^−/−^ progeroid and wildtype old mice, and restored their lymphoid vs*.* myeloid haematopoietic output (Zong et al., 2021). Restoring sirtuin activity in this way may be beneficial in immunosenescence, since antigen-experienced senescent CD8^+^CD28^−^ T cells, for example, have decreased SIRT1 expression compared to naïve T cells, which partly drives their glycolytic and cytotoxic aged phenotype (Jeng et al., 2018). It would be interesting to investigate whether NR supplementation may alleviate immunosenescence in other DNA repair-defective models, e.g., the *Vav-iCre* ^*+/−*^ *;Ercc1* ^*-fl/*^ mouse model (Yousefzadeh et al., 2021b).

The impact of NR supplementation on mitophagy is also accompanied by better DNA repair. In ATM-knockdown primary neurons treated with DNA-damaging etoposide, NR reduced 53BP1 staining (Fang et al., 2016). Mechanistically this was due to an increase in SIRT1-mediated deacetylation of Ku70 in the DNA-PK complex during DSB repair by NHEJ (Fang et al., 2016). Similarly, in *wrn-1(gk99)* mutant worms, NR treatment decreased the RAD51 signal 24 h after ionising radiation, this time suggesting an improvement in HR-mediated repair of DSBs (Fang et al., 2019). Accordingly, NR supplementation starting at 2 years-old in wildtype mice reduced the accumulation of γH2AX^+^ DNA-damaged muscle stem cells (Zhang et al., 2016). Taken together, these data suggest that NR may be genoprotective by improving both mitochondrial quality and DNA repair. We note that *in vitro* studies use relatively high concentrations of NR (0.5–1 mM) for improvement of DNA repair (Fang et al., 2016; Fang et al., 2019). Though current supplementation regimens in older adults robustly increase the NAD^+^ levels of peripheral immune cells, investigations into the effective doses of NR for *in vivo* genoprotection are needed (Martens et al., 2018).

#### 4.2.2 Enhancing DNA repair - enoxacin

We have discussed the evidence that persistent DNA damage is a primary driver of immunosenescence during ageing, and how existing interventions that ameliorate immunosenescence may be genoprotective by acting to promote DNA repair. Pharmacological interventions that target DNA repair pathways are common in the field of cancer chemotherapy, and often work to elicit cancer cell death by inhibiting DNA repair. However, drugs that act to promote DNA repair to elicit genoprotection have received less attention.

One exception is enoxacin, a fluoroquinolone antibiotic that boosts DNA repair by enhancing the RNAi machinery component, DICER, which increases the generation of DNA damage response non-coding RNAs (DDRNAs) (Gioia et al., 2019). Deficiency of the RNAi machinery impairs the DDR and increases genome instability (Swahari et al., 2016). However, treatment of cells with 50 µM enoxacin prior to ionising radiation caused a rapid accumulation of 53BP1 at DSBs which corresponded with a subsequent decrease in DNA lesions (as measured by comet assay and γH2AX levels) after the acute DDR, indicating a boost in overall DNA repair (Gioia et al., 2019). Thus, enoxacin appears to be genoprotective by enhancing DSB repair. In the context of ageing, enoxacin has been reported to increase *C. elegans* lifespan (Pinto et al., 2018), but little is known on its immune effects. It is of note that the SARS-CoV2 N-protein binds to DDRNAs, and by doing so outcompetes the 53BP1-DDRNA binding that is necessary for 53BP1 recruitment to DSBs (Gioia et al., 2023). In this way, SARS-CoV2 infection was recently demonstrated to increase DNA damage and induce cellular senescence, driving disease pathology (Gioia et al., 2023). It may be that enoxacin treatment, by increasing DDRNA expression, could promote 53BP1-mediated repair of DNA lesions, thus preventing senescence and potentially limiting morbidity and even mortality in COVID-19 patients. Most importantly, enoxacin is already well tolerated in older adults, and current treatment regimens increase enoxacin blood concentrations to a level relatively comparable to those used in *in vitro* studies for eliciting genoprotection (Table 2) (Hamel et al., 2000). Given the urgent call for novel drugs attenuating immunosenescence, future studies investigating enoxacin’s effect on DNA damage/repair in immune and non-immune cells, and on immune system function in older people, may be beneficial. More generally, we propose that genoprotective drug discovery campaigns may be a valuable approach towards attenuating immunosenescence.

## 5 Discussion

Improvements in global health have resulted in a steady increase in population lifespan over the past century. However, the widening gap between healthspan and lifespan has accelerated the need to uncover ageing mechanisms and interventions for promoting healthy ageing. DNA damage and repair play important roles in immunity and ageing: intentional induction of DNA lesions is crucial in the generation of a diverse immune repertoire, but poor resolution of DNA damage can induce a persistent DNA damage response that drives permanent exit from the cell cycle and senescence. Ageing is accompanied by an accumulation of senescent immune cells, exemplified by T cells, with persistent DNA damage that may directly cause ageing.

In this review we draw together evidence from the fields of DNA repair, ageing, and immunology, to explore why genome instability increases in old T cells and non-immune cells during ageing, and how this drives their maladaptive senescent phenotypes and promotes immunosenescence. We propose that existing geroprotective therapeutic approaches can limit DNA damage and promote DNA repair, and so we term these “genoprotective” interventions. We discuss potential genoprotective interventions, including dietary supplementation (vitamin D_3_ and NR), as well as treatments with repurposed drugs (metformin, mTOR inhibitors, p38 MAPK inhibitors), and finally a novel drug, enoxacin, that may boost DNA repair and which remains largely unexplored in longevity science. Overall, we suggest that investigations into improving DNA repair processes and genome stability, both for immune and non-immune cell types, are valuable strategies for targeting immunosenescence.
